# Supplementary material for: Are carbon emissions trading and green financial instruments synergistic? -Comprehensive quantitative research based on content analysis
Source: PLoS One. 2024 Mar 7;19(3):e0298601. doi: 10.1371/journal.pone.0298601 (PMC10919601; doi:10.1371/journal.pone.0298601)
Supplement: S1 Data — (ZIP) [file pone.0298601.s001.zip › Data/地级市Z_F工作报告.zip!/║┌┴·╜¡/─╡╡ñ╜¡╩╨/└┤╫╘╣1⁄2╓┌║┼í╛┬φ┐╦╩2╛▌═°í┐_2008.docx]

各位代表：

　　现在，我代表市人民政府向大会作工作报告，请予审议，并请各位政协委员和列席人员提出意见。

　　一、2007年工作回顾

　　2007年是本届政府任期第一年，也是全市追赶型跨越式发展起步之年。在省委、省政府和市委正确领导下，在市人大、市政协监督支持下，市政府团结和依靠全市各族人民，励精图治，锐意进取，奋力拼搏，攻坚克难，超额完成了市十四届人大一次会议确定的目标任务，是近年来改革创新力度最大、阶段性成效最明显、人民群众得实惠最多的一年，全市人民对未来充满希望、充满信心。

　　经济实现快速增长。预计地区生产总值实现420亿元，同比增长13%。财政实现“四个十”突破，全口径财政收入实现43.3亿元，增长32.8%，比上年纯增10亿元；市直财政收入突破10亿元，达到10.7亿元，增长30.1%；向上争取各类财政性资金26.2亿元，比上年增加10亿元；化解政府性债务10亿元以上。投资跳出低迷状态，全社会固定资产投资达到156.3亿元，增长54.4%。农业喜获丰收，粮食总产量达到348亿斤，增长0.7%，农村经济总收入实现174.2亿元，增长13.3%。工业主体地位和支撑作用更加突出，规模以上工业增加值、销售收入和税金实现54亿元、185亿元、10亿元，增长19.4%、18%和12%。内外贸易增势强劲，社会消费品零售总额实现157.9亿元，增长17.8%；外贸进出口总额达到74.4亿美元，增长20%，其中对俄贸易57亿美元，增长34%，占全省份额的53.7%、全国的13%。群众收入普遍增加，城镇居民人均可支配收入达到9251元，增长15.1%；农民人均纯收入达到5569元，增长18.8%，连续五年居全省首位。市区经济明显提速，县域经济增势强劲，绥芬河、东宁、海林跻身全省10强县，市县区联动、口岸与腹地互动的发展格局进一步的形成。

　　项目建设成效显著。整合区域资源，创新项目生成推进机制，120个投资5000万元以上市级重点项目开工建设，当年投资81.6亿元，项目开工数量和投资额均创历史最高。哈牡绥东对俄经济带新上项目65个，完成投资21.3亿元，其中牡丹江对俄经济开发区完成投资14亿元，13个项目开工建设、4个竣工投产，项目总数达到30个，总投资56亿元。中俄农业高新技术合作示范区、农产品出口加工园区和宁安农垦新城启动建设。万吨烧碱和1.5万吨树脂、2万吨醋酸乙烯、6000吨高档卷烟纸等支柱产业项目及三通、鑫鹏等农业产业化项目投产，油页岩、俄重油加工、荒沟电站、二发电四期等一批立市型大项目具备了开工条件，新引进天津宝迪等4个农业产业化项目，化工、农副产品加工、木业、能源等产业的集群效应开始显现。新增国家级高新技术企业5户、火炬计划重点企业2户、高新技术产品7项，镜泊湖牌绵白糖成为国家免检产品，三角牌运动弹获“中国名牌”称号。

　　改革开放向纵深推进。国企改革迈出新步伐，石油化工厂与首控战略合作开始启动，水泥集团与浙江卧龙集团完成股权重组，牡纺启动政策性破产，桦林集团、轴承厂政策性破产获国家批准。融资解债工作取得新突破，争取到省财政转移支付等9项政策。区域经济一体化进程明显加快，出台了资源整合、利益共享等11个方面激励政策，磨刀石、五林两镇划入市区，与农垦、森工、高校合作共建工作不断深化。各项改革取得新进展，行政审批事项由268项压缩到222项，全面推行了市、县（市）、区党政机关和事业单位公务用车货币化改革，完成了“四清”工作阶段性任务。对外开放取得新成效，国家商务部批复的康吉经贸合作区投入运营，跨国陆海联运大通道正式开通，开辟了我省新的出海口。组织参加“俄罗斯·中国年”、“哈洽会”等经贸活动，成功举办第三届中国（牡丹江）“木博会”、第二届中国（东宁）黑木耳节及全国小蘑菇新农村建设现场会，我市及东宁县、林口县、海林市分别被授予“中国食用菌之城”、“中国黑木耳第一县”、“中国食用菌优秀基地县”和“中国猴头菇之乡”称号。引进世茂、绿地、龙睿等一批战略投资项目，全市招商引资到位资金113亿元，增长51.5%，其中实际直接利用外资7581万美元，增长20.9%。全年承办各类会议会展600余个，其中国家级会议占1/4。接待国内外旅游者515万人次，实现旅游业总收入33.4亿元，均增长12%。

　　城乡面貌明显改观。全市总动员推进城乡建设，通过市场化运作有效破解资金瓶颈，总投资达到39亿元，其中城市建设投入是上年的7倍。东四跨江桥一期竣工通车，党政办公中心投入使用，江南新城区市政设施进一步完善。大力实施美化、绿化、亮化、净化工程，整修市区街路48条、桥梁4座，栽植花草树木120万株，评选出云杉、牡丹为市树、市花，集中开展了占道经营、私搭乱建、乱贴乱画等整治行动，灯饰化装点的广场、桥梁和楼体建筑物成为城市夜晚的靓丽景观。认真落实市人大1号议案，改造棚户区45万平方米，其中市区15.5万平方米。市污水处理厂恢复运营，海林、宁安污水处理厂开工建设。完成了年度减排任务，大气环境优良天数达到315天。新农村建设扎实推进，新建农村公路1118公里、安全饮水工程151处、乡镇客运站6个、农家店300个，组建农机作业合作社20个，新增沼气用户5500户，20户以上通电自然村实现了广播电视“村村通”，公路通达率、自来水普及率、住房砖瓦化率、电话和有线电视入户率均高于全省平均水平。镜泊湖水库除险加固等水利工程进展顺利，治理水土流失36.2万亩，造林绿化3.5万亩。

　　社会事业全面发展。首届牡丹江·镜泊湖之夏旅游文化节和雪城旅游文化节成功举办，打造出具有我市特色的旅游文化品牌。“同一首歌·走进牡丹江”等大型演出提高了对外影响力，“城市之光”系列活动丰富了市民文化生活。新创作出一批文艺精品，8项非物质文化遗产入选省级保护名录。全部免除了农村义务教育阶段学生学杂费，选派187名教师赴农村支教，现代化的一中新校区及六中东校区建成使用，师范学校、高级技工学校分别晋升为省幼儿专科学校和技师学院。农村新型合作医疗全面启动，参合率达到92%，乡镇卫生院改造达到70%，社区卫生服务覆盖率达到85%，整合了7所市属医疗机构，市妇儿医院新楼投入使用，第二社会福利院和慈善医院一期工程竣工。我市成为全省唯一的国家级婚育新风进万家示范市。驻牡军警部队广大官兵积极投身牡丹江经济建设和社会发展，特别是在完成急难险重任务中发挥了重要作用，双拥工作获全国“五连冠”。认真落实党的民族宗教政策，支持少数民族事业发展，东宁县列入全国少数民族“兴边富民”行动重点县。群众体育活动丰富多彩，竞技体育获国内外大赛金牌12枚。

　　发展成果惠及民众。社保扩面创历史最好水平，筹集社保资金18.4亿元，完成了11.4万名企业退休人员新增养老金发放工作，基本医疗保险参保人数达到40万人，基本医疗保险和大额医疗补助最高支付限额分别提高到3万元和17万元，17.1万名符合条件的城乡居民享受到低保补助，市区低保标准提高到月人均180元。通过制定专项政策、开展爱心资助等措施，为特困家庭落实廉租房200套，发放取暖补助金、廉租房租金补贴、助学款及减免医疗费用、提供商品价格救助近4000万元。多渠道开辟就业岗位，新增就业7.1万人，下岗失业人员再就业5.5万人，其中安置就业困难人员1.9万人，援助“零就业”家庭就业1155人，城镇登记失业率控制在3.7%以内。20件利民实事圆满完成。深入开展和谐社区示范活动，举办了首届“社区节”，社区服务功能进一步完善。

　　政府自身建设取得新成绩。大力推进政府“限权提效”、政务公开、政务信息化和诚信政府建设，建立了项目建设“四审联动”机制和部门协调联动机制，全面开展“治庸治懒”行动和“机关靓化”工程，加大了行政执法责任制落实力度，严肃查处了一批破坏环境、损害群众利益的违纪违法案件，机关效能建设得到加强。坚持向人大及其常委会报告工作和向政协通报情况制度，自觉接受法律监督和民主监督，办理人大议案、建议、批评和意见49件，政协提案206件，加强了与民主党派、工商联和人民团体的联系。开展了信访、安全生产和社会治安“百日攻坚战”，信访总量下降5.6%，安全生产事故下降34%，刑事案件破案率提高27.9%，安全度过防火期和防汛期。统计、物价、工商、质量技术监督、食品药品监督、国土资源管理、人事、接待、外事侨务、广播电视、人防、地震、气象、档案、地方志、老区、老龄、残疾人等工作都取得了新进步。

　　各位代表，过去一年的成绩来之不易，令人振奋，催人奋进。这些成绩，是市委正确领导的结果，是市人大、市政协大力支持的结果，是离退休老干部和社会各界关心、配合的结果，是全市上下自我加压、大胆创新、敢于碰硬、埋头苦干的结果。在此，我代表市人民政府，向辛勤工作在各条战线的全市人民，致以亲切的问候和崇高敬意！向为大牡丹江、新牡丹江建设做出积极贡献的中省直单位、驻牡部队和武警官兵，向大力支持政府工作的人大代表、政协委员和各民主党派、工商联、无党派人士、社会团体，向各位老领导、老同志，向所有外来投资者及关心支持牡丹江发展的国内外朋友，表示衷心的感谢！

　　过去一年的成绩，为做好今年工作、促进全市又好又快发展奠定了坚实基础。但我们也清醒地认识到，在加快发展进程中，一些矛盾和问题仍很突出：一是经济结构调整滞后，产业层次偏低，城市功能不全，加强环境保护和节约资源的任务越来越迫切；二是体制机制相对落后，市场发育和信用体系不健全，人才匮乏，开放度不够高；三是城乡困难家庭渴望得到更多帮助，人民群众渴望进一步提高收入水平、改善生活条件；四是各种历史矛盾和问题集中显现，财政进入还债高峰期，还债压力较大，信访和安全生产形势不容乐观，加强保稳定、保安全的基础工作和建立健全长效机制迫在眉睫；五是部分公务员的素质、能力和作风与发展的要求不相适应，政府的执行力、落实力和监督力亟待增强，服务环境亟待改善。与此同时，我们还看到，新征程中机遇与挑战并存，机遇大于挑战。一方面，国家提升沿边开放战略与俄罗斯开发远东地区互动，我国与韩国、日本经贸合作关系日益密切，更加凸显出我市的综合优势，增强了发展后劲；另一方面，全国各地纷纷掀起新一轮发展热潮，我市追赶跨越需要付出更大艰辛。所以，我们既要抢抓机遇，又要趋利避害；既要解放思想，又要真抓实干；既要抓好当前、速见成效，又要谋划长远、积攒后劲；既要毫不动摇地加快发展，又要安全至上、保护环境、节约资源；既要坚定不移地推进改革开放，又要妥善处理好各方面利益关系，维护和谐稳定的社会局面。只有这样，我们才能科学有效地做好各项工作，不负人民的重托。

　　二、2008年工作任务

　　今年是全面加快追赶型跨越式发展的攻坚年。我们要深入贯彻落实党的十七大精神，按照市委十届四次全会部署，坚持以科学发展观为统领，以追赶跨越和改善民生为主线，以改革开放为动力，突出工业立市、金融强市战略地位，强力推进经济带和大通道建设，狠抓招商引资、项目建设、园区经济和城市开发，进一步强化一产优势、提升二产竞争力、做大三产规模，协调发展各项事业，提高人民生活水平，促进社会和谐稳定，确保全市经济社会发展再上新台阶！

　　主要预期目标是：地区生产总值增长14%，达到500亿元。全口径财政收入增长20%，达到50亿元，力争保持30%左右的增幅。全社会固定资产投资增长35%，达到210亿元。农村经济总收入增长15%，达到200亿元。规模以上工业增加值、销售收入、税金增长23%、20%和15%。社会消费品零售总额增长16%，达到183亿元。外贸进出口总额增长25%，达到93亿美元，力争突破100亿美元。城镇居民人均可支配收入增长16%左右，达到10700元；农民人均纯收入增长15%，达到6400元。万元GDP综合能耗下降4.5%。为完成和超额完成上述目标，要切实做好以下七项工作。

　　（一）坚定不移推进工业立市战略，进一步做大做强工业经济。

　　全力抓好大项目建设。坚持“经济社会发展项目化”，开展“项目攻坚年”活动，依靠项目建设盘活存量、扩充增量、优化结构。强力推进“211”项目工程，重点抓好5000万元以上大项目200个，开工建设100个，完成投资100亿元，确保吉信木业、恒远药业等项目投产达效，富通空调、桦林佳通等扩产改造项目完成年度投资任务，荒沟电站、二道沟电站、二发电四期、东北高新“双十六”以及油页岩、俄重油加工等立市型大项目开工建设。提高园区和企业厂房容积率及项目投资强度，促进项目向集约化方向发展，牡丹江对俄经济开发区新开工项目15个，完成投资20亿元。完善项目生成、协调服务和考核奖惩机制，新谋划150个投资1000万元以上的项目。

　　重点扶持优势产业和企业。强化协调服务，着力帮助企业解决资金和铁路运力不足等问题，降低运行成本。扶强以12户骨干企业为重点的产业龙头，扶壮以20户“小巨人”为重点的创新型企业，优选100户具有发展潜力的成长型企业。做大造纸、能源、化工、汽车配套、木材加工5个支柱产业，改造提升轻纺、建材、医药、机械、农副产品加工等传统产业，培育生物工程、电子信息等新兴产业，形成梯队，放大总量，不断构筑工业经济发展的新优势。

　　深化企业改制重组。加快完成牡纺、桦林集团等5户企业政策性破产，妥善解决国企改制遗留问题，全面剥离企业水电汽及办社会职能，争取机床厂等一批企业改制重组，盘活存量资产20亿元。整合水泥、制药等企业，促进其向集团化方向发展。推动已改制企业二次重组、二次创业，鼓励企业搞活、扩张、做大，大力发展民营经济和股份制经济。组建外派监事会，强化国有企业外部监管，规范各类企业发展。

　　用高新技术改造传统企业。坚持提升与扩面并重，抓好恒丰纸业、金刚钻公司等企业信息化改造升级和北方阀业、双兴化工等企业信息化建设，完善镜泊啤酒等企业信息化系统集成，引导中小企业广泛应用信息技术。推进“质量兴市”，促进企业加强管理，增强自主创新能力，不断创品牌、创名牌。

　　大力发展循环经济。以循环经济试点园区为载体，发展环保、节能及废物利用等循环经济产业化项目，加快电石渣综合利用、粉煤灰免烧砖等项目建设。坚持“谁排污、谁付费，谁污染、谁治理”，严格落实节能减排责任，推进北方工具厂、二发电等10个废水处理和脱硫污染减排项目，抓好郭家沟垃圾填埋气发电和医疗废弃物无害化处理项目，城市规划区内全面禁用实心粘土砖。推进国家级海林板桥、东宁老黑山水电站“以电代燃”生态工程建设，整合水能、风能、矿产等资源和能源，谋划包装项目，高效开发利用。

　　（二）大力实施金融强市战略，进一步增强金融、财政和投融资的支撑作用。

　　在扶持企业上市上突破。把上市融资作为最有力的招商引资手段，制定三年上市规划，出台土地、金融、税收等扶持政策和奖励办法，建立“拟上市企业储备库”，加强上市辅导，创造上市条件，重点培育绥芬河控股、响水米业、石油机械等绩优企业，力争一年后有1－2家企业实现上市。

　　在金融产业发展上突破。积极为各商业银行发展创造条件，鼓励银行创新产品、拓展信贷领域、扩大信贷规模。壮大城市商业银行，深化农村信用社改革，探索建立农村新型金融组织。积极引进股份制银行及外资银行设立分支机构，扩大证券、基金、保险市场，发展委托贷款、风险投资等新的融资方式。提高广兴、鑫汇公司担保能力，规范发展民营担保公司，争取我市成为国家“三省一区”担保机构首批试点市，为中小企业融资创造条件。

　　在资产运作和化解债务上突破。树立经营财政理念，强化融资、解债、上市和资产运营“四位一体”平台建设，促进国有资产管理由静态管理向动态管理转变，由单纯管理向管理与经营并重转变，由资产管理向资本运作转变。加大向城投集团“三资”注入力度，做大做强融资平台，规范所属行业性公司运营模式。加快自来水公司等公益事业市场化步伐。扩大与长城、信达等四大国有资产经营公司的金融合作，提高化解债务能力。引进域外资金，组建新型担保公司、投资公司和资产管理公司，创新运营模式，提升资本运作能力。

　　在优化金融生态环境上突破。加强社会诚信体系建设，建立企业及个人信用档案，实行“黑名单”和公开暴光制度，制定失信惩戒办法。开展政府及法院、工商、税务、金融等部门联合行动，支持银行清收债务，严厉打击恶意逃废债务行为，建设金融安全区。完善政银企三方沟通协调机制，定期向金融部门通报政府重要决策、项目建设等情况，引导银行贷款向优势企业和重点项目倾斜。

　　在财源建设上突破。落实振兴老工业基地、财政贴息、科技三项费用及招商引资等政策和资金，扶持企业发展，加速重点项目向财源转化。增强国有资产控制力，通过资产资源参股、控股等方式，最大化地增加政府资产收益。进一步完善落实区域经济一体化政策，提升城区经济，壮大县域经济，发展共享经济，做大总部经济，鼓励飞地经济，培育结算经济。理顺市与区财政体制和税源级次，完善共享收入增收激励机制。坚持依法征税、科学征税、文明征税，强化税收征管，建立综合治税机构，对重点税源、零散税源进行全方位监控，确保应收尽收。

　　（三）加大改革开放力度，进一步增强经济社会发展活力。

　　坚持用改革的思路破解难题、推动发展。完善国库集中收付制度，实行非税收入管理改革，在“四清”基础上严格编制管理，加快行政事业单位资产集中管理和经营改革、国有资本经营预算改革，规范中介机构和各类收费，推进环卫、园林等公用事业体制改革以及教育、医疗、文化体制改革，推行供热货币化改革。加快农村土地流转，促进土地向规模经营集中，新流转土地30万亩。做好户籍制度改革试点工作，逐步实现城乡人口自由流动。实施统计调查体制和国民经济核算体系改革，推行“在地统计”，促进行政区经济向区域经济转变。

　　加快对俄对韩合作战略升级。以哈牡绥东对俄经济带和陆海联运大通道为载体，进一步探索创新，争取政策支持，创建国家提升沿边开放的先行区和示范区。围绕发展口岸经济、临港经济，加快落实绥芬河铁路口岸扩能改造，提高铁路运能，提高口岸过货能力、换运能力和通关效率。大力培育外贸主体，优化进出口结构，扩大对俄对韩出口，鼓励进口资源能源，积极拓展国际市场。扶持本地企业到境外从事资源加工、纺织服装、种养业和房地产开发。抓好中俄信息产业园、绥芬河海都机电城及对韩工业园、生态园建设。推进绥——波、东——波互贸区发展，完善双合果菜批发、浙中建材市场、亚洲商贸城等境内外市场，促进绥芬河远东工业园、康吉经贸合作区、华宇工业园区规模发展。拓展境外农业服务站功能，筹建海参崴商务联络机构，扶持企业“走出去”发展。争取恢复对俄航线，增加至首尔航班密度，开通至釜山等国际航线。

　　强力扩大招商引资和区域合作。以引进现代加工业、物流业、金融、旅游、房地产业战略投资者以及500强企业地区总部、研发机构、知名品牌为重点，精心组织好赴香港、韩国等重点目标市场招商活动，切实提高项目签约率、落地率，全年招商引资到位资金增长30%以上。深入开展跨区域合作，加大与上海、顺德等长三角、珠三角发达地区城市合作，规划辟建苏沪鲁工业园，制定扶持政策，主动承接发达地区产业转移。完善与农垦、森工及高校合作共建机制，启动一批农业产业化及旅游、教育、科研等合作项目。

　　切实增强创新能力。推进技术研发、资源共享、企业孵化、技术交易等创新平台建设，建立自主创新评价奖励制度，加强知识产权保护，支持自主知识产权产品实现产业化，促进以社会化、网络化为载体的科技服务体系建设。落实培养、引进、留住人才政策，完善柔性人才引进机制，建设牡丹江人才大市场，辟建创业园区和创业大厦，推进大学校区、科技园区、创业园区、公共社区“四区”融合，促进科研资源整合、各类人才集聚，构建区域创新体系，吸引“走出去”的牡丹江人回乡发展，欢迎海内外有识之士来牡丹江投资兴业。

　　（四）推进社会主义新农村建设，进一步壮大县域经济整体实力。

　　坚持项目向园区集中、农民向城镇集中、土地向规模经营集中，以农业产业化为基础，以城镇化为方向，优化农村经济结构。加快县域工业园区建设，提高产业集中度，促进县域工业向规模化、集约化发展。制定扶持政策，实施“突破林口”战略，推动各县（市）向全省十强和全国百强迈进。

　　用工业化理念发展特色农业。壮大肉牛、生猪、食用菌、果菜四个主导产业，扶持皓月、鑫鹏、正大、三通等龙头企业发展，确保兴达、宝迪、三鹿等企业开工投产。加快基地建设，力争肉牛、生猪分别出栏22万头、110万头，栽培食用菌12.7亿袋，优质瓜果菜种植面积达到100万亩，一品村发展到440个。完善农业标准化体系建设，无公害、绿色和有机食品认证面积达到460万亩。推进园区、企业、产业联动发展，启动省级外向型星火产业带建设，加快中俄高新技术合作示范园区、农产品出口加工园区和宁安农场农垦新城建设，规划建设100个种养业、休闲观光、农事体验等现代农业园区。整合优质资源，强化包装和市场营销，培育和叫响一批“牡丹江特产”。扩大境外农业开发规模，在俄种植面积达到220万亩，收益达到11亿元。

　　加强农村和农业基础设施建设。落实支农政策，开展帮建活动，高标准建设省、市、县（市）三级试点乡村，支持革命老区乡村发展，建设新生活、新家园、新制度、新风尚的“四新”型农村。开展“五清、五改、五化”整治活动，新建通村公路900公里，80%的行政村公路实现硬质化，新增安全饮水工程100处，新建“康居乡镇”5个、“康居新村”35个，完成40个村屯、150公里农村公路绿化，发展沼气用户4600户。改造完善瓜果菜等园艺设施，开工响水灌区一期工程，加快镜泊湖、九佛沟、清河、城子沟水库除险加固工程建设，保护母亲河，保养黑土地，以小流域为单元治理水土流失26万亩。加强耕地保护和土地复垦整理，抓好林业生态工程建设，植树造林2万亩，启动宁安10万亩省级林权制度改革试点。切实做好防汛、抗旱和森防工作。

　　完善农业社会化服务体系。推进农业信息入户示范工程，提高农村信息化水平。发挥新组建的牡丹江农业科学院作用，建立试验示范基地，带动新技术、新品种推广。落实农机补贴政策，壮大农机合作组织，田间作业综合机械化率达到81%。加强农产品质量检验检测中心、动物疫病防控和畜产品安全设施建设。加快发展农村经济合作组织，建立健全各类专业协会，壮大农村经纪人队伍，促进龙头企业和农户建立利益联结机制。推进“万村千乡市场工程”，新建农家店150个，发展农产品专业市场和大型综合配送集团。

　　大力培养新型农民。实施农民培训示范工程，整合培训资源，完善培训机制，促进技能型培训向素质型培训转变，深入开展科技、文化、医疗“三下乡”活动，组织农民到外地学习、开阔视野，着力培养一批有文化、懂技术、会经营的新型农民和农村致富带头人。做好农村劳动力转移工作，新转移农村劳动力1.1万人。

　　（五）加快发展现代服务业，进一步做大第三产业规模。

　　壮大旅游产业。突出“生态牡丹江、对俄大通道”主题，加强旅游宣传推介，扶持旅行社赴客源地开展外联促销活动，办好第二届牡丹江·镜泊湖之夏旅游文化节和雪城旅游文化节，创办东北虎文化节。开工和续建10个以上重点旅游项目，开辟避暑休闲度假游、冰雪休闲运动游、红色旅游和跨省跨国旅游精品线路，培育农业观光、工业生产、民俗风情以及文化、体育等旅游产品。推进镜泊湖晋升国家5A级景区工作，组建镜泊湖旅游公司，进一步整合资源，通过上市、项目融资等渠道，拓展发展空间，叫响镜泊湖、林海雪原、中国雪城、百年口岸和东北虎之乡等品牌。适应节假日调整变化，活跃城郊游、乡村游，满足市民的旅游需求。全年实现旅游业总收入40亿元，增长20%。

　　大力发展会展经济。坚持政府主导、企业主体、市场运作，制定会展业发展规划，鼓励发展会展公司，积极引进国内外会展企业，逐步实现由政府承办为主向市场运作为主转变。探索市里主办与县区申办相结合、市中心主会场与县区分会场相结合、市区与景区相结合等多种办会形式。办好第四届中国（牡丹江）“木博会”以及第三届中国（东宁）黑木耳节等活动，叫响“中国木业城”和“中国食用菌之城”等品牌。积极承办我省与俄远东地区经贸洽谈会，组织东北亚区域合作论坛、沿边地区市长论坛、太平洋地区经济会议，谋划创办新的会展活动。

　　培育新兴服务业。加快发展仓储配送、货运代理等现代物流业，启动华晟国运物流二期工程，引进有实力的物流合作伙伴，构建口岸过货物流、制造业物流和城市商品配送相结合的现代物流体系。规范发展会计、律师、公证以及资产评估、投资咨询、信用评级等中介服务业，积极发展现代房地产、服务外包和创意产业。建立健全各种要素市场，丰富时尚、休闲、保健等新兴业态。大力发展电子商务等信息服务业，整合现有各种信息平台，实现网络系统共建共享，推进“数字牡丹江”建设。

　　（六）深化和落实大牡丹江、新牡丹江战略构想，进一步加快城市化进程。

　　拉开城市框架。按照市委关于“三个重新”的要求，在更广阔的空间规划定位城市发展，开展城市总体规划修编工作，编制好城市概念规划、控详规划、工业用地规划和桦林、温春、铁岭、磨刀石四个分区规划，科学引领城市发展。坚持市场化运作为主、政府投入为辅，全年力争完成城乡建设投入75.5亿元。加快大牡丹江路网建设，续建长汀至双峰、宁安至杏山段公路、东宁老黑山至省界、林口至大罗密和牡丹江至宁安段公路，加快打通北太平路，争取启动牡绥铁路复线、牡绥高速公路建设，谋划北外环路、牡海宁城际公路，构建1小时经济圈。推进区划调整工作，合理进行县（市）村镇归并，尽快实现中心城市建成区100平方公里、人口100万人的“双百”目标。抓好绥芬河省级城乡一体化试点、东安区兴隆镇城市化试点和宁安农垦新城组团开发试点，完成兴隆镇改建制工作，构筑6县（市）城关镇和18个重点镇的城镇化体系，推动大牡丹江格局的进程。

　　优化提升中心城市功能。完善城市分区功能布局，实施“腾笼换鸟”、“优二进三”，推动江北老城区工业企业向园区和城郊集中，加快磨刀石镇、五林镇工业化与城镇化结合的步伐，启动四城区“城中村”改造试点。继续打造东安中央商务区、西安朝鲜民族集合区、阳明汽车贸易集中区、爱民大学服务区等特色街区。培育江南新城区以总部经济、结算经济为主的税收非物质化承载区及行政办公、文化教育和生态居住区。高起点、高标准建设城市基础设施，推进一批城市功能性大项目建设，完成城市规划展示馆建设，续建市客运枢纽站，积极推进机场跑道“盖被”、东四跨江桥二期、西十一条路跨江桥工程，异地建设市殡仪中心。实施“道路畅通”工程，新建和整修市区50条街路。

　　改善城乡人居环境。大力推进“三城同创”，加快建设生态型宜居城市。实施生态环保治理工程，加大中心城区绿化力度，新增绿地2万平方米，主城区植树14万株，推进西山公园、人民公园等6个公园改造提升，促进“一河三溪六湖”流域整治开发，加快4500米江滨景观带项目建设，打造城市新景观。创造条件，推进市污水处理厂二期和林海水库供水工程，加快海林、宁安污水处理厂建设，全面解决饮水安全问题。整顿关停小水泥、小木业等不达标企业，严格汽车尾气排放标准，完成锅炉并网100万平方米，进一步改善大气环境，倡导健康、节约、环保的消费模式。实施老城区楼房“穿衣戴帽”工程，完善80条小街巷路灯设施，开展居民小区楼道亮化和粉刷美化。大规模进行棚户区改造，改造面积比上年增长2倍以上。健全监管机制，防止“一房多售”，规范房地产市场。坚持建管结合，集中整治私搭乱建、乱贴乱画、占道经营、噪音扰民等行为，规范出租车、机动三轮车和人力车经营秩序。加强小区物业管理，推进达标升级。加快城市综合管理职能向江南新城区延伸，管理范围向城乡结合部拓展。

　　（七）高度关注民生，进一步解决好群众最关心的实际问题。

　　发展依靠人民，发展为了人民。我们要把今年作为“民生改善年”，通过多种渠道筹措资金23.7亿元，突出抓好8项惠民工程，办好50件实事，让人民共享改革发展成果。

　　以创业带动就业再就业。认真贯彻就业促进法，实施创业就业工程，出台全民创业实施意见，辟建市创业服务中心，投放小额担保贷款5000万元，鼓励扶持自谋职业、自主创业。坚持开发就业岗位与购买公益性岗位相结合，创新援助方式，重点解决“零就业”家庭、“4050”人员和城郊被征地农民就业问题，做好复员退伍士兵、大中专毕业生和城镇失业人员安置工作，创建“充分就业社区”，全年新增城镇就业6万人，安置下岗失业人员4万人。落实劳动合同法，开展劳动合同制度“三年行动计划”，加强劳动仲裁和执法监察，依法维护农民工和企业职工权益，建立与企业效益挂钩的从业人员工资增长机制和保障机制，年底前解决国有企业职工工资陈欠问题。

　　千方百计增强社会保障能力。继续扩大养老、失业、医疗、工伤和生育保险覆盖范围，争取列入全国城镇居民医疗保险试点城市，逐步将32万市区城镇居民纳入医保范围，取消农村医疗救助起付线，提高农村大病补贴最高限额，扩大城乡医疗救助病种，市区失业保险金标准提高20%，职工最低工资月标准提高到525元，城镇低保标准提高到月人均210元，农村低保标准提高到年人均800元，为1万名灵活就业人员发放社保补贴，提高企业退休人员养老金水平。制定出台被征地农民社会保障办法。实施安居工程，完善住房保障体系，推进廉租房保障制度由低保家庭向低收入住房困难家庭延伸，为300户“双困”家庭解决廉租房，为100户农村贫困残疾家庭改造危房。

　　不断提高群众健康水平。实施医疗保障工程，有效解决群众“看病贵、看病难”问题。实现新型农村合作医疗制度全面覆盖，参合农民达到实有农民的93%以上。改造和建设1所县医院、4所乡镇卫生院，建成46所社区卫生服务机构，社区卫生服务覆盖率达到100%。推进20个重点医疗专科建设，提高对重大传染病和突发公共卫生事件的监测、预警和应急处置能力。完善农村药品供应和监督网络，确保农民用药安全、质优价廉。积极推进二院搬迁、中医院扩建工程，建设慈善医院二期和爱心家园。提高出生人口素质，落实农村计划生育家庭奖励政策。积极发展妇女儿童事业、老年人事业以及心理健康、残疾人康复事业，推进“居家养老”和“爱心护理”工程。

　　全力帮扶困难群体。实施帮困救助工程，深入开展困难职工、失业人员、低保人员等困难群体帮扶活动，投入600万元对市区低保家庭和困难职工实施价格救助，通过“爱心救助超市”救助困难群众2.5万人次。壮大志愿者、义工规模，新建爱心服务基地10个，广泛开展助困、助老、助残活动，受益群众达到3万人次。整合社会福利院、儿童福利院和流浪儿童保护中心，建设社会福利家园。实施爱心助学工程，建立全省低保家庭学生免费学历教育基地，通过爱心助学、希望工程、彩虹工程、春蕾计划，资助1万名贫困学生就学，决不让一个学生因贫困失学。

　　办好人民满意的教育。实施大教育发展战略，坚持公益性投入与市场化运作相结合，有效整合优化教育资源，鼓励和规范社会力量办学，建成市幼教中心，组建职业教育集团，启动江南基础教育示范园和大学城建设，组建牡丹江大学绥芬河学院。完善义务教育经费保障机制，落实城乡义务教育免除学杂费政策，抓好农村寄宿制学校建设，改造市五中、九中等学校基础设施，不断改善城乡办学条件。强化教育信息化平台建设，促进城乡教育资源共享，建设学习型社会。推进以课程改革为重点的素质教育，加强教师队伍建设，抓好学生思想品德教育，保障“流动生”、“留守生”健康成长。办好少数民族教育，关心支持特殊教育。

　　丰富城乡居民精神文化生活。加强爱国主义基地建设，实施博物馆、八女英烈纪念馆、牡丹江烈士馆、杨松纪念馆“四馆合一”工程。加强文化基础设施和文艺创作队伍建设，鼓励多出文艺精品。加强文物保护工作，挖掘非物质文化遗产。加强文化市场管理，开展以网吧、出版物为重点的文化市场整治行动，净化文化市场环境。引导民营资本进入文化领域，形成文化产业多元投入机制，鼓励发展戏剧、歌舞、影视、动漫等文化产业，规划建设创意产业园，把旅游、体育、商贸、城建等行业与文化产业发展有机结合起来，加快大文化产业发展进程。着力建设专业运动员队伍，提高竞技体育水平。实施文体益民工程，开展丰富多彩的城乡文化活动和“全民健身与奥运同行”系列活动，为农民和农民工免费放映电影5600场，建设射击馆、轮滑场和40处城乡健身设施。

　　扎实推进“平安牡丹江、和谐牡丹江”建设。严格执行信访工作责任制、领导包案制和信访责任追究制，认真落实市长接待日制度，建立群体性突发事件协调机制、矛盾排查调处机制和社会稳定风险预测评估机制，发挥人民调解、行政调解、司法调解联动作用，控制与疏导相结合，依法治访，和谐解访，切实解决好信访渠道反映出的实际问题。吸取血的教训，狠抓安全管理，健全安全防范、预警长效机制和应急救援体系，强化对各类安全隐患的排查整改，坚决关闭不达标的小煤矿和非煤矿山，坚决遏制重特大安全事故。加强市场监管，严惩食品药品生产流通过程的制假售假等行为。采取有效措施平抑物价，防止搭车涨价、哄抬物价。举办第二届社区节，进一步完善社区管理服务体系。广泛开展以平安家庭、社区、乡镇、县（市）为重点的和谐创建活动，深入实施社区和农村警务战略，依法加强宗教事务管理，严厉打击各种刑事犯罪和邪教组织，增强人民群众的安全感。强化边境管理控制，保持边境地区的良好秩序。

　　三、切实加强政府自身建设

　　打铁必须自身硬。我们要适应新形势、新任务要求，进一步增强责任意识、忧患意识和创新意识，进一步提高凝聚力、战斗力和执行力，更好地肩负起加快追赶型跨越式发展的重任。

　　（一）强化依法行政，建设法治政府。扎实推进依法治市，深入开展“五五普法”，营造自觉学法守法用法的社会氛围。认真贯彻国务院《全面推进依法行政实施纲要》，完善依法行政责任体系，强化行政执法责任制，规范行政执法，自觉依法办事，严查各种执法违法行为。实行政府法律顾问制度，完善重大决策专家咨询论证及听证、公示等制度，保证各项决策的科学性、民主性。落实《政府信息公开条例》，加快电子政务建设，全面推行以阳光执法、阳光审批、阳光服务和新闻发言人制度为重点的政务公开制度，完善“民心网”、市长信箱和市长热线，进一步提高政府工作透明度，最大限度地保障人民群众的知情权。重大事项决策前及时向市委和市人大常委会报告，将政治协商纳入政府决策程序，认真办理人大议案、建议、批评和意见及政协提案，主动接受人大依法监督、政协民主监督和社会舆论监督。支持工会、共青团、妇联等社会团体开展工作。

　　（二）强化作风建设，建设责任政府。深入推进“治庸、治懒、治散”专项行动和机关“靓化工程”，坚持“严”字当头，严格要求，严明责任，严肃管理，进一步把各级干部的心思、精力和行动集中到追赶型跨越式发展的实践中来。大兴学习之风，促进各级干部认真学习中国特色社会主义理论和党的十七大精神，潜心钻研经济、金融、法律和业务知识，培养一批“发展型”干部和“项目型”人才，增强各级干部的科学发展能力、社会管理能力、抓落实能力和协同配合能力。大兴调研之风，沉下去研究工作、谋划项目、解决问题，察民情、排民忧、解民困。大兴求真务实之风，报实情、说实话、办实事，坚决防止弄虚作假、报喜不报忧。掀起“责任风暴”，实行更加严格的督办检查、更加严格的绩效评估、更加严格的责任追究，真正做到对人民负责、让人民满意。

　　（三）强化职能转变，建设服务政府。以强化公共服务职能为重点，以优化经济发展环境为突破口，进一步加快政府职能转变，把政府工作重心转到社会管理和公共服务上来。教育干部站在投资者、创业者和人民公仆的角度切身体验、换位思考，自觉增强服务意识，切实解决教条执法、机械执法等问题。优化政府工作流程，继续简化审批事项、缩短审批时限，落实好绿色通道、全程代理、领导包办等工作机制，努力为投资者、企业和市民服好务，打造零障碍、低成本、高效率的兴业安商环境。进一步规范行政收费，严格限制对企业进行评比、检查和各种变相摊派行为。改革行风评议制度，改进评议办法，建立定期征询机制，重点对公众关注程度高的行政执法部门进行行风暗访，公开评议结果，对暴露出来的中梗阻、潜规则以及不作为、乱作为等损害环境的行为严厉查处，绝不姑息。

　　（四）强化监督力度，建设廉洁政府。坚持教育、制度、监督并重，毫不放松地抓好廉政建设，切实形成艰苦创业、干净做事的良好风气。加强反腐败教育和廉政文化建设，切实增强各级干部的廉政意识和抗腐蚀能力，严格执行廉洁自律各项规定。认真落实党风廉政建设责任制，健全行政行为约束机制，充分发挥监察和审计部门的职能作用，做到用制度管权、按制度办事、靠制度管人。坚决纠正吃拿卡要等不正之风，强化商业贿赂专项治理，依法查处项目规划审批、工程建设招投标、政府采购、土地出让、产权交易等重点领域和环节的腐败行为。带头加强诚信建设，以政府诚信带动社会诚信。开展创建节约型机关活动，降低行政成本，反对奢侈浪费。

　　各位代表！实现市委提出的全面加快追赶型跨越式发展的任务光荣而艰巨。让我们在市委的坚强领导下，紧紧依靠全市人民，坚定信心，埋头苦干，开拓进取，奋勇争先，加快建设经济大市、开放大市、品牌大市，努力创造牡丹江人民幸福美好的新生活！
